# Seroepidemiological surveillance, community perceptions and associated risk factors of malaria exposure among forest-goers in Northeastern Thailand

**DOI:** 10.3389/fcimb.2022.953585

**Published:** 2022-08-22

**Authors:** Mohd Amirul Fitri A. Rahim, Sriwipa Chuangchaiya, Paisit Chanpum, Laun Palawong, Panuwat Kantee, Nor Diyana Dian, Inke Nadia D. Lubis, Paul C. S. Divis, Akira Kaneko, Kevin K. A. Tetteh, Zulkarnain Md Idris

**Affiliations:** ^1^ Deparment of Parasitology and Medical Entomology, Faculty of Medicine, Universiti Kebangsaan Malaysia, Kuala Lumpur, Malaysia; ^2^ Faculty of Public Health, Kasetsart University, Chalermphrakiat Sakon Nakhon Province Campus, Sakon Nakhon, Thailand; ^3^ Vector Borne Disease Unit, Ban Koh Sub-District Health Promoting Hospital, Mukdahan, Thailand; ^4^ Department of Paediatric, Faculty of Medicine, Universitas Sumatera Utara, Medan, Indonesia; ^5^ Malaria Research Centre, Faculty of Medicine and Health Sciences, Universiti Malaysia Sarawak, Kota Samarahan, Sarawak, Malaysia; ^6^ Department of Microbiology, Tumor and Cell Biology, Karolinska Institutet, Stockholm, Sweden; ^7^ Department of Parasitology, Graduate School of Medicine, Osaka Metropolitan University, Osaka, Japan; ^8^ Department of Infection Biology, Faculty of Infectious Tropical Diseases, London School of Hygiene and Tropical Medicine, London, United Kingdom

**Keywords:** malaria, serology, *Plasmodium falciparum*, *Plasmodium vivax*, thailand

## Abstract

Malaria remains a major public health challenge in Thailand. Continuous assessment and understanding of the behavior and perceptions related to malaria exposure in the high-risk group are necessary to achieve the elimination goal. This study aimed to investigate the parasite prevalence, seroprevalence rate, knowledge, attitudes, and practices (KAP), and malaria risk factors in rural communities living close to a forested area in the northeastern part of Thailand. A community-based cross-sectional survey was conducted in three forest-goer communities (i.e., Ban Khok, Ban Koh, and Dong Yang) located in Khamcha-i district, Mukdahan Province, Thailand, from July to August 2019. Demographic, socioeconomic information and KAP data were collected using a structured questionnaire. Parasite prevalence was determined by microscopy. Seroprevalence was determined *via* ELISA using two *Plasmodium falciparum* (PfAMA-1 and PfMSP-1_19_) and two *Plasmodium vivax* (PvAMA-1 and PvMSP-1_19_) antigens. Age-adjusted antibody responses were analyzed using a reversible catalytic model to calculate seroconversion rate (SCR). Malaria parasite was not detected in any of the 345 participants. The overall malaria seroprevalence was 72.2% for PfAMA-1, 18.8% for PfMSP-1_19_, 32.5% for PvAMA-1, and 4.4% for PvMSP-1_19_. The proportion of seroprevalence for *P. falciparum* and *P. vivax* antigens was significantly highest in Ban Koh (35.1%, *P* < 0.001) and Don Yang (18.8%, *P* < 0.001), respectively. For all parasite antigens except PvMSP-1_19_, the proportion of seropositive individuals significantly increased with age (*P* < 0.001). Based on the SCRs, there was a higher level of *P. falciparum* transmission than *P. vivax*. Regarding KAP, almost all respondents showed adequate knowledge and awareness about malaria. Nevertheless, significant effort is needed to improve positive attitudes and practices concerning malaria prevention measures. Multivariate regression analyses showed that living in Ban Koh was associated with both *P. falciparum* (adjusted odds ratio [aOR] 12.87, *P* < 0.001) and *P. vivax* (aOR 9.78, *P* < 0.001) seropositivities. We also found significant associations between age and seropositivity against *P. falciparum* and *P. vivax* antigens. The data suggest that seroepidemiological surveillance using AMA-1 and MSP-1_19_ antigens may provide further evidence to reconstruct malaria exposure history. The absence of weak evidence of recent malaria transmission in Mukdahan Province is promising in the context of the disease elimination program.

## Introduction

Malaria is a vector-borne disease transmitted to humans by the infectious bites of female *Anopheles* mosquitoes. Globally, malaria is the top mosquito-borne disease that causes infection and death. In the World Health Organization (WHO) Southeast Asia region, approximately 200 million people in nine countries are at high risk of acquiring malaria (WHO, 2020a). Thailand has made significant progress toward eliminating malaria by 2024 (WHO, 2020b). Over the last 5 years, the country has continuously reduced its malaria burden; cases have decreased by 84%, from 34,611 in 2014 to 5,425 in 2019 (Ministry of Public Health, 2019). However, this elimination effort still needs to be strengthened in the context of the surveillance system as a key intervention strategy to accelerate the progress toward the elimination target.

Although malaria control efforts in Thailand have been highly effective in decreasing infection, malaria is still endemic and a common disease in some areas of Thailand. The spread of malaria in Thailand varies and can be characterized as “forest malaria” and “border malaria” with high transmission along international borders, including Myanmar, Cambodia, Laos, and Malaysia (Sattabongkot et al., 2018). The distribution of malaria along the borders accounted for 69.5% of malaria cases, with the highest prevalence on the western border with Myanmar (Sriwichai et al., 2017). Moreover, there has been a migration of laborers from Myanmar to the Thai-Cambodian border provinces, mainly from high-transmission areas (Parker et al., 2015). In a retrospective study, the border regions of Thailand-Myanmar and Thailand-Cambodia, with high numbers of migrant workers, were found to have the highest malaria incidence rates, including *Plasmodium falciparum*, *Plasmodium vivax*, and mixed infection (Imwong et al., 2015). Several studies also documented naturally acquired human infections with non-human primate malaria in areas near the border with Malaysia (Putaporntip et al., 2009; Jongwutiwes et al., 2011; Ngernna et al., 2019), thus serving as warning signs of an increasing zoonotic malaria burden in Thailand.

Previous studies in Thailand have shown that malaria is widespread among demographic groups engaged in certain high-risk behaviors (Cotter et al., 2013; Ly et al., 2017). For example, in Northeast Thailand, the most vulnerable group to malaria includes those living in remote areas, mobile individuals, and migrant populations (Delacollette et al., 2009). These populations also live and work in areas with high malaria transmission and high human-vector contacts, such as forests and forest-fringe areas. They are mostly illiterate, deprived, and poorly connected with public health and surveillance programs, hospitals, and legitimate pharmacists (Parker et al., 2015; Munajat et al., 2021). They are more likely to seek treatment from unregulated, private vendors who may raise their risk of exposure to non-standard and counterfeit medications or artemisinin-based monotherapies (Yeung et al., 2008). Their high mobility makes health promotion messages by the health authority challenging to reach. As a result, newcomers from non-endemic areas to endemic areas are at greater risk of infection because they have not been exposed to educational and prevention measures directed at reducing risk (Ly et al., 2017). Therefore, it is crucial to inform or remind the whole community to adopt preventive measures to reduce the risk of infections.

The primary objective of this cross-sectional study was to determine malaria (*P. falciparum* and *P. vivax*) antibody responses in an asymptomatic population of forest-goer communities living in rural areas in Mukdahan Province, one of Thailand’s upper northeastern provinces. Three endpoints were assessed to characterize malaria burden in the study population: (i) malaria seroprevalence of individuals measured by detection of antibodies to *P. falciparum* and *P. vivax*, (ii) knowledge, attitudes, and practices (KAP) regarding malaria prevention and control, and (iii) potential risk factors associated with malaria seropositivity.

## Materials and methods

### Study sites

**Figure 1:**
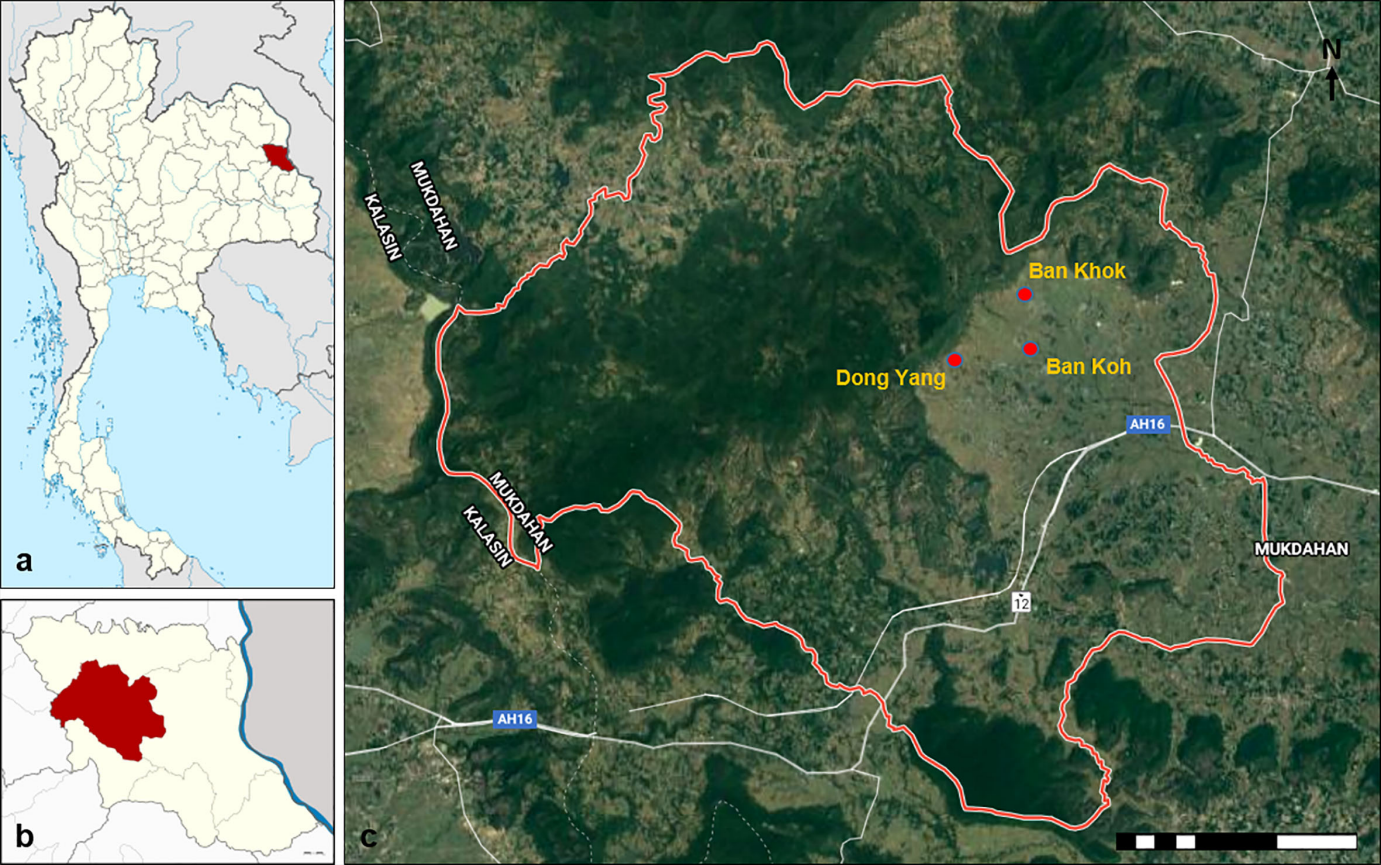
Map of the study area. **(A)** Map of Thailand showing the location of Mukdahan Province (red). **(B)** Map of Mukdahan Province showing the location of Khamcha-i district (red). **(C)** The location of the three study villages within the Khamcha-i district.

The study was conducted in three villages, namely, Ban Koh (latitude 16.5798°N; longitude 102.4791°E), Ban Khok (latitude 28.0024°N; longitude 202.0825°E), and Dong Yang (latitude 15.7539°N; longitude 103.2721°E) located at the Khamcha-i district of Mukdahan Province, Northeastern Thailand (**Figure 1**). Ban Koh, Ban Khok, and Dong Yang have a population of approximately 453, 646, and 708, respectively (Ministry of Interior, 2019). They were selected based on the recommendation from the local District Health Office, aged 7 years or older, representativeness of typical forest-fringe villages, availability of water bodies for breeding of *Anopheles* mosquitoes, and ongoing traditional activity of forest foraging involving frequent close contact with wild macaques. Mukdahan Province is located in the country’s northern region (i.e., Isan region). It had a population size of 342,200 in 2019 (Ministry of Interior, 2019). To the east, it borders the Mekong River, across which lies the Savannakhet Province of Lao People’s Democratic Republic (Lao PDR). West of the province is the Phu Phan mountain range, covered with thick dry dipterocarp and evergreen forests, including several national parks and other protected areas. This mountain range is also home to two non-human primate populations, namely, the rhesus macaque (*Macaca mulatta*) and the pig-tailed macaque (*Macaca nemesterina*) (Srikosamatara and Doungkhae, 1982). The typical climate of the area is tropical, with an average annual temperature of 27°C and an average annual rainfall of 1,524 mm. There is a short dry season between January and April, followed by a long wet season from May to December. The main economic activity is based on agriculture, such as wet rice cultivation. The villagers also relied on rubber tapping and gathering and selling forest products for their livelihood.

In 2013, 14 malaria cases were reported in the Khamcha-i district, including one fatal case due to vivax malaria (unpublished data, Department of Disease Control of Ministry of Health in Mukdahan Province). Furthermore, the incidence of malaria is generally higher in Mukdahan Province due to its location along the international border of Savannakhet Province in Lao PDR and relatively porous, with a high potential for malaria importation. Among the 18 provinces of Lao PDR, Savannakhet has the third highest malaria incidence (Phommasone et al., 2016). A recent malaria survey in Savannakhet reported 20% asymptomatic *Plasmodium* infections, of which 11.1% and 3.6% were due to *P. vivax* and *P. falciparum*, respectively (Phommasone et al., 2016). In addition, the large degree of population movement, particularly for people and migrants who visit forested areas where residual malaria transmission continues, represents a major challenge to malaria control in this region (Chang et al., 2021; Jongdeepaisal et al., 2022).

### Study design and data collection

A cross-sectional survey was carried out between July and August 2019. All Thai nationals of both genders and residing in the study villages were eligible. A convenience sampling strategy was used, whereby residents were asked to come to the selected survey points such as temples or clinics. Participants were briefed on the study procedure, and their consent was documented. Individuals who could not communicate or were unwilling to provide informed consent were excluded. Furthermore, axillary body temperature was also measured using a digital thermometer (Terumo, USA), and those with temperatures exceeding 37.5°C were considered febrile.

A standardized questionnaire was developed and administered to each participant to gather data on demographic, socioeconomic status and KAPs on malaria. The questionnaire included a mixture of closed-ended and open-ended questions. A pre-tested and validated pilot survey was conducted in communities in other settings to evaluate the questionnaire’s clarity, adequacy, and comprehensibility. In addition, preliminary interviews were conducted to ensure standard interviewer methods and relevant interview questions were used. The interviews were conducted anonymously by trained interviewers with on-site supervision to minimize the courtesy bias and improve the accuracy of participants’ self-reporting. Before conducting the interviews, the lead researchers (SC and ZMI) conducted a one-day training for the surveyors to explain each question and how to conduct the interviews. The interview was conducted face to face using the Thai language, and all questions were open-ended. A knowledge score was calculated according to the correct answers.

### Parasite detection by microscopy

A blood sample was obtained by finger prick for thick and thin blood smears, and three spots of blood (approximately 100 µl per spot) were collected on Whatman ET31 Chr filter paper (Whatman, UK). All blood smears were stained with Giemsa solution and examined for the presence of malaria parasites under a 100X high-power field by experienced microscopists as described previously (Idris et al., 2016; Idris et al., 2017a). Blood spots on filter paper were air-dried and stored in plastic bags at 4°C for the short term and −20°C for the long term.

### Serological assays

Four 3-mm disks were punched from each dried blood spot, and serum was eluted in reconstitution buffer in 0.5-ml deep-well plates (Thermo Fisher Scientific, USA) as described previously (Corran et al., 2008; Idris et al., 2017a; Idris et al., 2017b). The reconstituted blood spot solution, equivalent to a 1/200 dilution of serum, was stored at −20°C until used for an antibody test.

All sera were tested for IgG antibodies by an indirect quantitative enzyme-linked immunosorbent assay (ELISA) to recombinant apical membrane antigen-1 (AMA-1) and 19-kDa fragment of recombinant merozoite surface antigen-1 (MSP-1_19_), i.e., *P. falciparum* (PfAMA-1 and PfMSP-1_19_) and *P. vivax* (PvAMA-1 and PvMSP-1_19_), as described previously (Idris et al., 2017b). Briefly, antigens were coated on Immulon 4 HBX plates (Thermo Scientific, USA) at concentrations of 2 µg/ml, 2.37 µg/ml, 0.86 µg/ml, and 1.55 µg/ml in coating buffer (50 µl per well) for PfAMA-1, PfMSP-1_19_, PvAMA-1, and PvMSP-1_19_, respectively. The plates were washed in phosphate-buffered saline (PBS) with 0.05% Tween 20 (PBS/T) and blocked using 1% (w/v) skimmed milk solution (Sigma, USA) in PBS/T for 3 hours. After washing, 50 µl of reconstituted blood spot solution was added in duplicate at final dilutions of 1:1,000 for both MSP-1_19_ antigens and 1:2,000 for AMA-1 and incubated overnight at 4°C. A positive control consisting of a pool of hyper-immune sera (i.e. pooled sera from infected humans) was included in each plate. The plates were washed, and 50 µl of horse-radish peroxidase (HRP)–conjugated rabbit anti-human IgG antibody (Elabscience, USA) were added to all wells at a dilution of 1:15,000 in PBS/T and incubated for 3 hours. After further washes, 100 µl of the substrate solution 3,3′,5,5′-tetramethylbenzidine (TMB) (Elabscience, USA) was added. Reactions were stopped after 15 min with 100 µl per well of 0.3M H_2_SO_4_. The optical density (OD) at 450 nm was read using a Multiskan Go ELISA reader (Thermo Scientific, USA).

### Statistical analysis

The data collected were tabulated into an Excel spreadsheet (Microsoft, USA) and cross-checked for errors. Data were processed and analyzed using Stata/SE version 13.1 for Windows (StataCorp, TX, USA). Data were presented as frequencies and percentages for categorical variables, whereas data for continuous variables were presented as the median and interquartile range (IQR). Duplicate ELISA OD values were averaged and normalized against values from blank wells to adjust for background reactivity (Corran et al., 2008; Idris et al., 2017a; Idris et al., 2017b). Seropositivity was determined by fitting a mixture model to normalized OD values assuming two Gaussian distributions, one for seronegative individuals and another for seropositive individuals (Idris et al., 2017a; Idris et al., 2017b). The mean OD + three standard deviations associated with the seronegative group was used as the cutoff value for seropositivity. A separate cutoff was generated for each antigen. The titer of antibody responses was estimated using the equation: dilution/[maximum OD/(OD test serum − minimum OD) − 1]. Differences in antibody responses were assessed using age-adjusted linear regression. Seroprevalence was stratified into yearly age groups and then analyzed using a reverse catalytic modelling approach under a binomial sampling assumption, as described elsewhere (Drakeley et al., 2005; Corran et al., 2007). This method provides estimates of the mean annual rates of conversion to seropositivity (seroconversion rate, SCR [λ]) and reversion to seronegativity (seroreversion rate, SRR [ρ]), averaged over the age of the population. Factors associated with *P. falciparum* and *P. vivax* seropositivities were determined for each site separately using generalized estimating equations adjusting for correlation between observations from the same variables. Gender, setting, age group, occupation, and income level were considered as explanatory variables in the univariate analysis. The reference group for the logistic regression analysis was based on the lowest seroprevalence of any *P. falciparum*– and *P. vivax*–specific antigens in each variable. Variables that were significant at *P* < 0.10 in the univariate analyses were added to the multivariate model and retained in the final multivariate model if their association with immune responses was statistically significant at *P* < 0.05.

## Results

### Sociodemographic characteristics and parasite detection

A total of 345 respondents in Mukdahan Province participated in this study (**Table 1**). Except for Ban Khok where sampling was conducted in a local clinic, respondents in Ban Kho and Dong Yang were sampled in their village temples. Among respondents, 38.6% were inhabitants of Ban Khok, 21.7% of Ban Koh, and 39.7% of Dong Yang. A slight majority of respondents were females (51.7%), and gender distributions were similar across the three villages (*P* = 0.212). The median age of the respondents was 57 years (IQR: 47–64). More than 90% of the respondents were over 30 years. At enrollment, the mean axillary temperature of the population differed across the three villages (*P* < 0.001), but no febrile illness (axillary temperature > 37.5°C) was recorded. Majority of the respondents were farmers (94.2%) and most had a monthly household income of Thai Baht (THB) ≤8,000 (~$260). There was no evidence of recent malaria transmission in the study areas, as evidenced by negative microscopy results in the three studied villages.

**Table 1:** Sociodemographic characteristics of respondents in three forest-goer communities in Mukdahan Province, Northeastern Thailand in 2019.

Characteristics	Overall	Ban Khok	Ban Koh	Dong Yang	*P*-value
Total number of respondents, n (%)	345 (100)	133 (38.6)	75 (21.7)	137 (39.7)	–
Gender, n (%)					
Male	166 (48.1)	70 (52.6)	38 (50.7)	58 (42.3)	0.212
Female	179 (51.9)	63 (47.4)	37 (49.3)	79 (57.7)	
Age, median (IQR), years	57 (47-64)	54 (42-62)	57 (48-64)	58 (50-65)	0.072
Age group, n (%), years					
7–15	15 (4.4)	6 (4.5)	2 (2.7)	7 (5.1)	0.058
16–30	16 (4.6)	9 (6.7)	6 (8)	1 (0.7)	
31–45	49 (14.2)	26 (19.6)	6 (8)	17 (12.4)	
46–60	147 (42.6)	52 (39.1)	33 (44)	62 (45.3)	
> 60	118 (34.2)	40 (30.1)	28 (37.3)	50 (36.5)	
Axillary temperature, median (IQR), °C	36.1 ± 0.5	36.4 (36.1–36.6)	36.3 (36.1–36.5)	35.9 (35.7–36.2)	<0.001
Temperature >37.5°C at time of survey, n (%)					
No	345 (100)	133 (100)	75 (100)	137 (100)	–
Yes	0 (0)	0 (0)	0 (0)	0 (0)	
Occupation, n (%)					
Farmer	325 (94.2)	126 (94.7)	70 (93.4)	129 (94.2)	0.182
Student	13 (3.8)	2 (1.5)	4 (5.3)	7 (5.1)	
Government	7 (2)	5 (3.8)	1 (1.3)	1 (0.7)	
Income level^*^, n (%)					
≤THB 8,000	303 (91.3)	122 (93.1)	62 (87.3)	119 (91.5)	0.366
>THB 8,001	29 (8.7)	9 (6.9)	9 (12.7)	11 (8.5)	
Malaria microscopy, n (%)					
Negative	345 (100)	133 (100)	75 (100)	137 (100)	–
Positive	0 (0)	0 (0)	0 (0)	0 (0)	

^*^No income recorded for 13 respondents.

IQR, Interquartile range; THB, Thai Bhat.

### KAP regarding malaria by area

Generally, almost all respondents (97.7%) were knowledgeable about malaria transmission *via* mosquito bites (**Table 2**). Approximately one-fifth of the respondents (20.9%) believed that malaria could be transmitted by drinking water in the forest, with significantly highest number of respondents from Dong Yang (*P* < 0.001). However, 96% of the respondents recognized that fever, headache, and chill were symptoms of malaria, with significant differences between study sites (*P* < 0.001). A small proportion of respondents (3.8%) reported not taking any medication after contracting malaria. Respondents from Ban Khok showed the highest proportions of knowledge about drug resistance due to incomplete adherence to the antimalarial drug (97.7%, *P* < 0.001), using bed net to prevent malaria (100%, *P*<0.001), and the importance of malaria examination after returning from the forest (100%). However, they were lowest on the knowledge of relapsing fever due to malaria (13.5%, *P* < 0.001).

**Table 2:** Knowledge, attitudes, and practices (KAP) on malaria among respondents in three forest-goer communities in Mukdahan Province, Northeastern Thailand in 2019.

Variables	Overall	Ban Khok	Ban Koh	Dong Yang	*P*-value
**Knowledge about malaria**					
Do you know that malaria caused by mosquito bites? n (%)					
No	8 (2.3)	6 (4.5)	1 (1.3)	1 (0.7)	0.099
Yes	337 (97.7)	127 (95.5)	74 (98.7)	136 (99.3)	
Do you think if you drink water from the forest you can easily get infected by malaria? n (%)					
No	273 (79.1)	124 (93.2)	57 (76)	92 (67.2)	<0.001^*^
Yes	72 (20.9)	9 (6.8)	18 (24)	45 (32.8)	
Do you know that malaria can cause fever, headache and chill? n (%)					
No	15 (4.3)	2 (1.5)	12 (16)	1 (0.7)	<0.001^*^
Yes	330 (95.7)	131 (98.5)	63 (84)	136 (99.3)	
If you get infected with malaria, do you take any medication? n (%)					
No	13 (3.8)	1 (0.8)	4 (5.3)	8 (5.8)	0.063
Yes	332 (96.2)	132 (99.2)	71 (94.7)	129 (94.2)	
Do you know you can develop drug resistance if you are not complete the antimalarial drug? n (%)					
No	35 (10.1)	3 (2.3)	3 (4)	29 (21.2)	<0.001^*^
Yes	310 (89.9)	130 (97.7)	72 (96)	108 (78.8)	
Do you know the use of a bed net can prevent malaria infection? n (%)					
No	19 (5.5)	0 (0)	5 (6.7)	14 (10.2)	0.001^*^
Yes	326 (94.5)	133 (100)	70 (93.3)	123 (89.8)	
Do you think you need to examine yourself from malaria after coming back from the forest? n (%)					
No	3 (0.9)	0 (0)	3 (4)	0 (0)	–
Yes	342 (99.1)	133 (100)	72 (96)	137 (100)	
Do you think if you get infected with malaria, you may have a relapsing fever later? n (%)					
No	215 (62.3)	115 (86.5)	1 (1.3)	99 (72.3)	<0.001^*^
Yes	130 (37.7)	18 (13.5)	74 (98.7)	38 (27.7)	
**Attitude toward malaria transmission**	** **	** **	** **	** **	
Live within 500 m from the forest, n (%)					
No	93 (26.9)	25 (18.8)	27 (36)	41 (29.9)	0.015^*^
Yes	252 (73.1)	108 (81.2)	48 (64)	96 (70.1)	
Spending the night in the forest within the last 6 months, n (%)					
No	261 (75.7)	112 (84.2)	54 (72)	95 (69.3)	0.011^*^
Yes	84 (24.3)	21 (15.8)	21 (28)	42 (30.7)	
Visited the nearby forest within the last 6 months, n (%)					
No	107 (31.1)	38 (28.6)	26 (34.7)	43 (31.4)	0.632
Yes	238 (68.9)	95 (71.4)	49 (65.3)	94 (68.6)	
**Practice to prevent the spread of malaria**	** **	** **	** **	** **	
Bed net ownership, n (%)					
No	261 (75.7)	49 (36.8)	75 (100)	137 (100)	–
Yes	84 (24.3)	84 (63.2)	0 (0)	0 (0)	
Sleep under a bed net^†^, n (%)					
No	0 (0)	0 (0)	0 (0)	0 (0)	–
Yes	84 (100)	84 (100)	0 (0)	0 (0)	
IRS within the last 12 months, n (%)					
No	0 (0)	0 (0)	0 (0)	0 (0)	–
Yes	345 (100)	133 (100)	75 (100)	137 (100)	
The practice of managing the illness, n (%)					
Go to the nearby clinic immediately	201 (58.3)	4 (3)	74 (98.7)	123 (89.8)	<0.001^*^
Wait out the symptoms until well	133 (38.5)	124 (93.2)	1 (1.3)	8 (5.8)	<0.001^*^
Purchase medication from the local shop	11 (3.2)	5 (3.8)	0 (0)	6 (4.4)	0.198

*Significant difference P < 0.05.

†Among those who own bed net (n = 84).

IRS, indoor residual spraying.

Concerning attitudes, most respondents (73.1%), with significant predominance from Ban Khok (81.2%, *P* = 0.015), reported living within 500 m of the forest. Although the villagers live near a forested area, respondents from Don Yang (30.7%, *P* = 0.011) and Ban Khok (71.4%) reported the highest proportions of spending the night and entering the forest for the past 6 months, respectively. Most respondents (75.7%) did not own bed nets as part of the practice to prevent the spread of malaria; however, only respondents in Ban Khok responded well to this practice (63.2%). Nevertheless, there was remarkable compliance for nightly bed net usage among those who owned them (100%). Similarly, all respondents reported 100% indoor residual spraying (IRS) coverage by the health authority at least once during 2019. Moreover, approximately 60% of the respondents reported seeking immediate treatment at the nearby clinic when malaria symptoms developed, with the significantly highest number of respondents from Ban Koh (*P* < 0.001). In contrast, respondents from Ban Khok claimed the highest proportion of waiting out the symptoms until well (93.2%, *P* < 0.001). Only a small proportion still considered buying medication directly from the local shop (3.2%).

### Breadth antibody responses and seroprevalence

**Figure 2:**
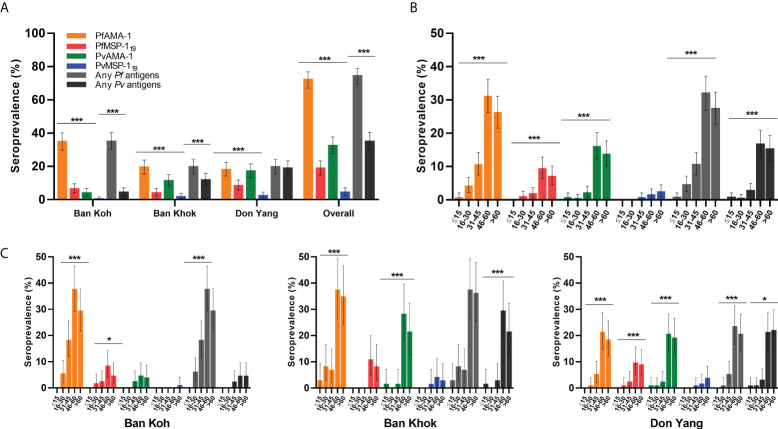
Specific seroprevalence of *P. falciparum* (PfAMA-1 and PfMSP-1_19_) and *P. vivax* (PvAMA-1 and PvMSP-1_19_) by the study area and age group. **(A)** Area-specific seroprevalence of participants by the study area. **(B)** Overall age-specific seroprevalence of participants and **(C)** age-specific seroprevalence of the study area. Seroprevalence is defined as the proportion of seropositive individuals in the study population based on a specific cutoff value generated for each recombinant antigen. Differences between the groups were determined using the Chi-square test or Fisher’s exact test. *P < 0.05; ** *P* < 0.01, ***P < 0.001.

Antibody responses to PfAMA-1, PfMSP-1_19_, PvAMA-1, and PvMSP-1_19_ were evaluated as OD levels, and antibody titers are shown in **Table 3**. Except for median PfMSP-1_19_ antibody OD level, median antibody OD levels and titers of malaria antigens varied significantly by study sites (*P* < 0.001). **Figure 2** shows the overall seroprevalence against parasite antigens in Mukdahan Province. Overall, malaria seroprevalence was 72.2% for PfAMA-1, 18.8% for PfMSP-1_19_, 32.5% for PvAMA-1, 4.4% for PvMSP-1_19_, 79% for any *P. falciparum* antigens, and 40.4% for any *P. vivax* antigens. Between the study areas, the proportion of seropositivity for any *P. falciparum* and *P. vivax* antigens was significantly highest in Ban Koh (35.1%, *P* < 0.001) and Don Yang (18.8%, *P* < 0.001), respectively. Within the study areas, the proportion of PfAMA-1 was significantly higher than other parasite antigens (all *P* < 0.001), ranging from 45.3% to 90.2%. For all parasite antigens, except for PvMSP-1_19_, the proportion of seropositive individuals significantly increased with age (all *P* < 0.001). Similarly, most parasite antigens showed a similar trend in the age-specific proportion of seropositivity within the study areas, i.e., highest in the 46–60 age group and lowest in the ≤16 age group.

**Table 3:** Malaria species–specific antibody responses and seroconversion rates.

Category^†^	Characteristic	Overall	Ban Khok	Ban Koh	Dong Yang	*P*-value
PfAMA-1	Median OD (IQR)	0.54 (0.34–0.72)	0.61 (0.48–0.74)	0.77 (0.54–0.99)	0.31 (0.21–0.49)	<0.001*
	Median antibody titer (IQR)	88 (49–164)	72 (46–97)	58 (34–110)	167 (92–326)	<0.001*
PfMSP-1_19_	Median OD (IQR)	0.29 (0.19–0.43)	0.31 (0.21–0.43)	0.29 (0.20–0.41)	0.29 (0.19–0.44)	0.827
	Median antibody titer (IQR)	103 (66–186)	98 (62–157)	89 (55–158)	131 (75–260)	0.001*
PvAMA-1	Median OD (IQR)	0.86 (0.54–1.19)	0.64 (0.42–0.86)	1.08 (0.74–1.34)	0.97 (0.66–1.27)	<0.001*
	Median antibody titer (IQR)	1,236 (493–2,623)	582 (340–11,834)	2,018 (960–3,695)	1,624 (848–3,092)	<0.001*
PvMSP-1_19_	Median OD (IQR)	0.52 (0.41–0.62)	0.47 (0.35–0.56)	0.55 (0.44–0.67)	0.54 (0.44–0.64)	<0.001*
	Median antibody titer (IQR)	691 (462–1,059)	498 (325–732)	883 (566–1,308)	855 (609–1,215)	<0.001*

*Significant difference P < 0.05.

†Calculated cutoff value for each recombinant antigen: PfAMA-1 = 0.343, PfMSP-1_19_ = 0.475, PvAMA-1 = 1.058, and PvMSP-1_19_ = 0.856. OD, optical density; IQR, interquartile range (25th–75th percentile).

### Malaria seroconversion rate

**Figure 3:**
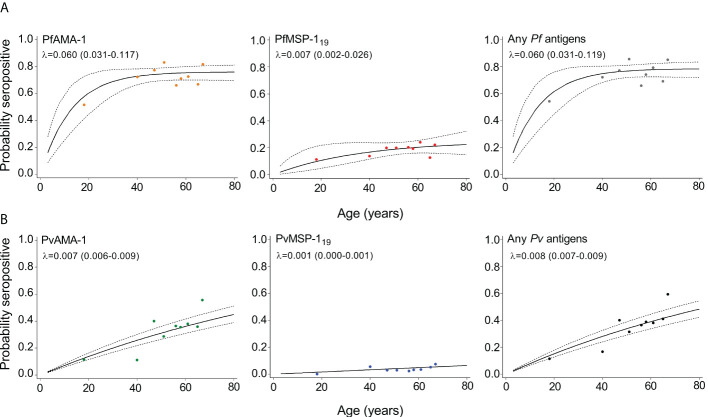
Annual probability of malaria seroconversion rate in Mukdahan Province. **(A)** *P. falciparum–*specific antigens and **(B)** *P. vivax*–specific antigens by age in each setting. Points indicated observed seroprevalence, and solid lines show model-predicted seroprevalence. Broken lines represent 95% confidence intervals. Seroconversion rates (SCR; λ) are presented on the graph.

The annual probability of malaria seroconversion curves in Mukdahan Province is shown in **Figure 3**. Based on the SCRs, there was a higher level of *P. falciparum* transmission than *P. vivax* using all tested antigens, ranging from 0.007 year^−1^ (0.002–0.026) to 0.060 year^–1^ (0.031–0.119) for *P. falciparum* and 0.001 year^−1^ (0.000–0.001) to 0.008 year^-1^ (0.007–0.009) for *P. vivax*. The SCRs were statistically significant between species-specific antigens, evidenced by the non-overlapping confidence intervals.

### Factors associated with seropositivity

Univariate and multivariate logistic regression analyses to identify factors associated with seropositivity to any *P. falciparum*– and *P. vivax*–specific antigens are shown in **Table 4**. Significant associations were found between setting and seropositivity in the adjusted model for any *P. falciparum*– and *P. vivax*–specific antigens. For *P. falciparum* antigens, a significant association was observed in Ban Koh (adjusted odds ratio [aOR] 12.9, *P* < 0.001) and Ban Khok (aOR 15.6, *P* < 0.001). In contrast, for *P. vivax* antigens, the increased seropositivity was significant in Ban Koh (aOR 9.4, *P* < 0.001) and Don Yang (aOR 6.9, *P* < 0.001). Interestingly, the likelihood of seropositivity increased significantly with age for only *P. falciparum* antigens (all *P* < 0.001), whereas for *P. vivax* antigens, the increase in seropositivity was not significant with age (all *P* > 0.05). No association was found between gender, occupation, income level, and seropositivity to any *P. falciparum*– and *P. vivax*–specific antigens.

**Table 4:** Logistic regression analyses of seropositivity to any *P. falciparum*– and *P. vivax*–specific antigens in Mukdahan Province, Northeastern Thailand.

Variable	Category	*P. falciparum* seropositivity (PfAMA-1 + PfMSP-1_19_)						*P. vivax* seropositivity (PvAMA-1 + PvMSP-1_19_)				
		n (%)	Crude OR (95%CI)	*P-*value	Adjusted OR (95%CI)	*P-*value		n (%)	Crude OR (95%CI)	*P-*value	Adjusted OR (95%CI)	*P-*value
Gender	Male	121 (72.9)	1.00		1.00			53 (31.9)	1.00		1.00	
	Female	136 (75.9)	1.18 (0.72–1.91)	0.511	1.55 (0.87–2.74)	0.136		68 (37.9)	1.31 (0.84–2.04)	0.239	1.17 (0.72–1.89)	0.530
Setting	Ban Koh	68 (90.7)	10.23 (5.18–20.22)	<0.001*	12.87 (5.23–31.71)	<0.001*		41 (54.7)	9.78 (4.69–19.17)	<0.001*	9.41 (4.56–19.29)	<0.001*
	Ban Khok	121 (90.9)	9.86 (4.22–22.99)	<0.001*	15.63 (7.26–33.65)	<0.001*		15 (11.3)	1.00		1.00	
	Dong Yang	68 (49.6)	1.00		1.00			65 (47.5)	7.10 (3.77–13.38)	<0.001*	6.85 (3.59–13.03)	<0.001*
Age group	7–15	2 (13.3)	1.00		1.00			2 (13.3)	2.31 (0.19–28.47)	0.514	1.33 (0.10–17.31)	0.824
	16–30	15 (93.8)	97.49 (7.91–1,202.91)	<0.001*	129.62 (8.89–1,888.77)	<0.001*		1 (6.3)	1.00		1.00	
	31–45	36 (73.5)	17.99 (3.57–90.79)	<0.0018	41.19 (6.44–263.43)	<0.001*		9 (18.4)	3.37 (0.39–28.96)	0.267	2.45 (0.28–21.79)	0.420
	46–60	110 (74.8)	19.32 (4.17–89.65)	<0.0018	67.49 (11.32–402.29)	<0.001*		57 (38.8)	9.49 (1.22–73.89)	0.031*	6.32 (0.79–50.49)	0.082
	> 60	94 (79.7)	25.46 (5.38–120.51)	<0.0018	94.85 (15.56–578.09)	<0.001*		52 (44.1)	11.82 (1.51–92.41)	0.019*	7.96 (0.99–63.99)	0.051
Occupation	Farmer	246 (75.7)	3.63 (1.19–11.13)	0.024*	0.40 (0.08–2.11)	0.281		119 (36.6)	3.18 (0.69–14.58)	0.137	1.14 (0.19–6.75)	0.886
	Student	6 (45.2)	1.00		1.00			2 (15.4)	1.00	1.00		
	Government	5 (71.4)	2.92 (0.41–20.89)	0.287	0.19 (0.01–2.71)	0.221		0 (0)	–	–	–	
Income level	≤THB 8,000	230 (75.9)	1.20 (0.51–2.82)	0.676	1.31 (0.50–3.39)	0.588		112 (36.9)	1.84 (0.76–4.45)	0.174	2.13 (0.83–5.46)	0.117
	>THB 8,000	21 (72.4)	1.00		1.00			7 (24.1)	1.00		1.00	

Odd ratios (ODs) and their 95% confidence intervals (95% CI) are presented for both univariate (crude) and multivariate (adjusted) models. Statistical significance was determined using the likelihood ratio test.

^*^Significant difference P < 0.05. n = number of seropositive.

OR, Odd ratio; THB, Thai Bhat.

## Discussion

This study is the first report on malaria seroprevalence for two *Plasmodium* species (*P. falciparum* and *P. vivax*): insights into KAP and risk factors of malaria exposure among forest-goer communities in rural areas of Mukdahan Province, Thailand. The results showed an apparent heterogeneity of antibody responses for AMA-1 and MSP-1_19_ antigens of both species, with AMA-1 demonstrating a higher prevalence of 4- to 8-folds compared to MSP-1_19_. The relationship between age and seroprevalence rates was similar in all studied areas, increasing with age, peaking at 46–60 years, then decreasing with age. In addition, seroprevalence and SCRs were higher in the older age group, indicating that the age-specific antibody response reflects cumulative exposure or population behavioral differences (San et al., 2022).

This study used serological approaches to measure malaria transmission metrics for serosurveillance purposes. Overall results show that humoral antibodies against *P. falciparum* antigens were higher than *P. vivax* antigens. This finding is consistent with previous studies in low transmission settings in Tak Province in Northwestern Thailand (Baum et al., 2016) and other areas in Southeast Asia, namely, Indonesia (Surendra et al., 2019), Myanmar (Edwards et al., 2021), and Vietnam (San et al., 2022). The dominance in exposure to *P. falciparum* could be attributed to the timing of data collection, which mainly occurred after the transmission season. It may also be attributed to the fact that the serological assay was initially designed to detect responses to *P. falciparum* antigens, thus was more optimal in detecting this species (Tadesse et al., 2017; Björkman et al., 2019; Edwards et al., 2021). Furthermore, *P. falciparum* circulates at higher parasite densities, which create higher antibody levels than *P. vivax*, and this is expected to induce more detectable antibody levels (Cook et al., 2012; Edwards et al., 2021). Moreover, antibodies against *P. falciparum* accumulate and degrade slowly over time, whereas antibodies against *P. vivax* are acquired quickly but are less durable (Yap et al., 2020). Further research could improve *P. vivax* antibody detection by running the assay at higher serum concentrations against more antigenic targets.

The community data on SCR using the tested antigen describes the differences in transmission intensity of *P. falciparum* and *P. vivax* in Mukdahan Province. Based on antigen stratification, AMA-1 has higher SCR than MSP-1_19_ for both species. Similar observations of higher SCR for AMA-1 than MSP-1_19_ were reported in other previous seroepidemiological studies (Cook et al., 2010; Tusting et al., 2014; Wong et al., 2014; Idris et al., 2017a; Idris et al., 2017b; Kwenti et al., 2017; Surendra et al., 2019; van den Hoogen et al., 2020). Differences in malaria exposure or transmission estimates between the AMA-1 and MSP-1_19_ may be linked to differences in seroconversion and reversion rates, which could explain the differences observed in our study. The different antigen seroconversion and reversion rates between the two antigens are possibly due to differences in their inherent immunogenicity, subclass-dependent half-life, and polymorphism. In addition, AMA-1 appears to be more immunogenic than MSP-1_19_, with higher anti-AMA-1 titers, implying that seroconversion and seroreversion for AMA-1 may be faster than MSP-1_19_ (Stewart et al., 2009; Idris et al., 2017a).

In this study, knowledge of malaria transmission was high among forest-goers in Mukdahan Province. Almost all respondents (98%) acknowledged that mosquito bite transmits the malaria parasite to humans, and this is consistent with the reported 100% bed net use by those who owned them. A comprehensive systematic review by Nofal et al. reported that forest-goer respondents in the Greater Mekong Subregion (GMS) countries, including Thailand, often described that mosquito bites were the cause of malaria (Nofal et al., 2019). Nevertheless, we noted a misconception regarding the transmission mode, whereby about a fifth of the respondents believed that drinking unclean water could also transmit malaria. In GMS countries, misconceptions are common, such as beliefs that malaria can be transmitted *via* bathing in contaminated water, exposure to contaminated wind, tiredness, poor hygiene, and eating specific dishes (Nofal et al., 2019). In addition, among forest-goers in Cambodia, supernatural deities and forest spirits were also reported as malaria causes (Lim et al., 2017; Pell et al., 2017; Verschuere et al., 2017).

Local variation in attitude toward malaria transmission was also observed among communities in the province. Almost a quarter of the respondents spent the night in the forest within the last 6 months, but it varied between settings (i.e., 16%–31%). According to a Cambodian report, forest-goers spent a maximum of a week per month in the forest, and the malaria risk varied among different communities (Kunkel et al., 2021). Furthermore, this study also found that significantly higher respondents from 31 to 60 years frequented the forest. As a working-age population, they are responsible for their family economy and thus strive to gain additional income through forest activities (Bannister-Tyrrell et al., 2019; Nofal et al., 2019; Kunkel et al., 2021). In addition, some foresters stay longer in the forest to increase their earnings, especially during harvesting periods (Canavati et al., 2019). This behavior increases the risk of malaria among forest-goers. Therefore, a comprehensive epidemiological study is needed to understand better the risk factors associated with seasonal exposure and time spent in the forest in this community.

The application and full coverage of long-lasting insecticidal nets (LLINs)/insecticide-treated bed nets (ITNs) are effective methods for malaria prevention and control (WHO, 2017). Accordingly, the target of the Ministry of Public Health of Thailand is one bed net per two persons in active foci areas of the country (Bureau of Vector-borne Disease, 2019). Although the present study found no evidence of recent transmission as indicated by no parasite-carrying individuals detected, a varying degree of community conformity to prevent malaria by bed net application was apparent. Overall, there was a low number of mosquito bed net ownership (24%) in the communities and, surprisingly, from only one village (i.e., Ban Khok, 63%). These results are consistent with the finding among the general population of Thailand that showed low individual ownership and coverage of bed nets (Kitidamrongsuk et al., 2016; Pooseesod et al., 2021). The stark differences in bed nets ownership between villages in the present study may be due to access to government health facilities. As the only clinic is located in Ban Khok, residents of Ban Khok can easily request bed nets compared to those from Ban Kho and Dong Yang. The other reason might be due to the insufficient number of bed nets obtained from the local health manager and operational challenges of the bed nets distribution system, as some studies identified operational barriers to bed nets distribution (Webster et al., 2013; Theiss-Nyland et al., 2017). This study suggests that the performance of the bed nets distribution system and the operational challenges of the bed nets distribution in Thailand should be evaluated. Notable failures of the local malaria control program thus include low net coverage by the health authorities and non-adherence to bed net use by the communities. The local health should also engage the community directly through education and awareness programs on the importance of mosquito bed nets.

Nearly 42% of respondents did not go to the clinic or health facilities when they experienced malaria-like symptoms. It may be due to the remote location of the villages, causing difficulty for villagers in obtaining health assistance. Also, the problem of getting public transport is a factor contributing to the low percentage of those who seek medical treatment in health facilities. A study in Myanmar also reported that the long distance between villagers’ houses and local health facilities was a factor in the community’s reluctance to seek malaria treatment (Than et al., 2019). Furthermore, most respondents who did not seek medical attention are over 30 years old; they probably relied on traditional healers or self-medication practices to treat malaria. A previous study conducted at the Thailand-Myanmar border of Tak Province showed that most respondents initially attempted self-treatment when they developed malaria symptoms, and some visited a traditional healer (Sonkong et al., 2015). In addition, some respondents think that the cost of diagnosis and treatment of malaria is expensive, so they choose to only buy medicine at the store without consulting qualified health care personnel. Self-awareness of malaria symptoms needs to be increased to encourage the villagers to seek medical treatment in health facilities. Government and non-government sectors should also increase their efforts to provide essential health care personnel for all villages, especially those in remote and endemic areas.

Multivariable analyses in each study area identified factors associated with malaria seropositivity. The finding concluded that there is a significant relationship between settings and antibody responses to *Plasmodium* species (*P. falciparum* or *P. vivax*). The risk of seropositivity for *P. falciparum* and *P. vivax* was discovered in Ban Khok and Ban Koh, respectively, which corresponded to higher adjusted odd ratios. Moreover, it was interesting to note that the increasing age of the population is a risk factor for *P. falciparum* exposure but not for *P. vivax*. It may be due to differences in risk behavior, such as occupational activity involving forestation or agricultural activities, which increase their exposure to malaria vectors between the older and younger age groups. In addition, *P. vivax* seroprevalence was extremely low; therefore, the lack of any correlation was most likely a result of statistical restrictions due to the small number of seropositive samples. Another possibility is that *P. vivax* infections cause lower antibody responses or shorter-lived responses not detectable by the current assay (Surendra et al., 2019; Tayipto et al., 2022).

This study has some limitations: (i) insufficient sample size for statistical analysis. Although small sample sizes may be adequate to detect a significant decline in SCR, they inevitably contribute to poor estimation precision of current SCR and restrict the possibility of identifying substantial changes in malaria transmission over time for the reverse catalytic model. (ii) While the convenient sampling was efficient and cost-effective, it has inherent selection bias. The survey was conducted mainly during the weekdays; thus, the younger adults were disproportionally represented. This group of individuals is typically away from home during weekdays, and under-representing them may likely underestimate the actual data. (iii) The assessment was based on a questionnaire, thus not representing the participants’ actual behavior. (iv) The cross-sectional study design provides information about a specific point in time, and the response of participants may not be the same in different seasons. Thus, we are unable to determine rates of change or stochastic variation. (v) The validity of the information provided by respondents may be questionable in the absence of independent checks or attempts to minimize errors of recall; however, this limitation is difficult to overcome due to inadequate funding.

## Conclusions

This study provides critical insight into the seroepidemiological surveillance of malaria in low transmission settings in Mukdahan Province, Northeastern Thailand. The present study also demonstrated that history of exposure can be constructed in the situation of missing baseline data and predictions can be made in the absence of active transmission. Furthermore, although most populations living in the study area have an acceptable level of knowledge about malaria, positive attitudes and practices concerning managing malaria require marked improvement. Finally, given the relatively low ownership of bed net, there is a need for future studies to engage the community directly through education and awareness programs on the importance of mosquito bed net. Continued malaria surveillance, vector control, education, and information campaigns are important not only among the forest-goer community but also among other vulnerable communities in the country.

## Data availability statement

The raw data supporting the conclusions of this article will be made available by the authors, without undue reservation.

## Ethics statement

The studies involving human participants were reviewed and approved by Ministry of Health of Mukdahan Province, Thailand (ref. no.101.0032.2562) and the Medical Ethics Committee, National University of Malaysia (ref. no. JEP-2019-148). Written informed consent to participate in this study was provided by the participants’ legal guardian/next of kin.

## Author contributions

ZMI, SC, INDL, and PCSV conceived and designed the study. MAFAR, SC, PC, LP, PK, NDD, and ZMI performed the fieldwork. AK and KKAT expressed proteins. MAFAR, SC and NDD performed data cleaning and analyzed the data. MAFAR, SC, INDL, PCSV, AK, KKAT, and ZMI interpreted the data. MAFAR, SC, and NDD wrote the first draft of the manuscript. ZMI, INDL, PCSV, AK, and KKAT provided critical revision of the manuscript for important intellectual content. All authors have contributed to the final version of the manuscript.

## Funding

This work (P.I: ZMI) was supported by the ASEAN Science Technology and Innovation Fund (ASTIF; FF-2019-124) from the ASEAN Secretariat and Geran Pembiayaan Sepadan (FF-2019-124/1) from the Faculty of Medicine, Universiti Kebangsaan Malaysia.

## Acknowledgments

We would like to extend our gratitude to the communities and community leaders for their support and participation in the survey. We wish to sincerely thank all members of the field team. We are grateful to the Khamcha-i District Public Health Office director and the director of Sub-District Health Promoting Hospital at Ban Koh for their support throughout the study. We are grateful to Thiti Srihanam, Sudarat Chaisangrat and Sujitrapa Surasan for their assistance in the survey. We sincerely thank Professor Rahmah Noordin for her critical reading of the manuscript.

## Conflict of interest

The authors declare that the research was conducted in the absence of any commercial or financial relationships that could be construed as a potential conflict of interest.

## Publisher’s note

All claims expressed in this article are solely those of the authors and do not necessarily represent those of their affiliated organizations, or those of the publisher, the editors and the reviewers. Any product that may be evaluated in this article, or claim that may be made by its manufacturer, is not guaranteed or endorsed by the publisher.
